# Transcatheter Closure of Atrial Septal Defect in a Patient with Cor Triatriatum Sinister and Atrial Septal Defect

**DOI:** 10.1155/2011/740981

**Published:** 2011-08-01

**Authors:** Wasana Hongkan, Kriangsak Thongchaiprasit, Kritvikrom Durongpisitkul

**Affiliations:** ^1^Division of Cardiology, Department of Pediatrics, Chonburi Hospital, Chonburi 20000, Thailand; ^2^Division of Cardiology, Department of Pediatrics, Faculty of Medicine, Siriraj Hospital, Mahidol University, Bangkok 10700, Thailand

## Abstract

Cor triatriatum sinister is a rare congenital heart disease and rarely found in adults. The authors describe an asymptomatic 20-year old man presenting with heart murmur by check up. Transthoracic and transesophageal echocardiogram demonstrate atrial septal defect (ASD) secundum 26 mm and cor triatriatum sinister with 20 mm of fenestration. Transcatheter closure of ASD using Occlutech Figulla^R^ device was successfully performed without complications. Echocardiogram post procedure demonstrate good position of device without obstruction of blood flow, no residual shunt and residual 12 mm of fenestration of cor triatriatum.

## 1. Introduction

Cor triatriatum sinister is a rare congenital heart disease with incidence of about 0.1–0.4% [1]. It is defined as fibromuscular membrane that divides left atrium into posterior-superior-positioned proximal cavity and anterior-inferior-positioned distal cavity [2]. The two cavities communicate through fenestration of the membrane. 

Atrial septal defect or patent foramen ovale was present in 70–80% of patients with cor triatriatum [3–5]. The clinical symptoms depend on the size of fenestration of the membrane. If the fenestration is restrictive lit will cause left-sided obstructive heart physiology with elevated left atrial pressure and associated hemodynamic consequences such as pulmonary congestion in neonatal and infancy period.

Appearance in adulthood is rare and usually associates with multiple or large fenestration that is presenting with asymptomatic cases [6–8].

Indication for ASD closure in adult is right atrial and right ventricular dilatation by echocardiography, CT, or MRI with one or more of the following: ASD diameter > 10 mm on echocardiography or/and Qp : Qs > 1.5 : 1 [9]. 

**Figure 1:**
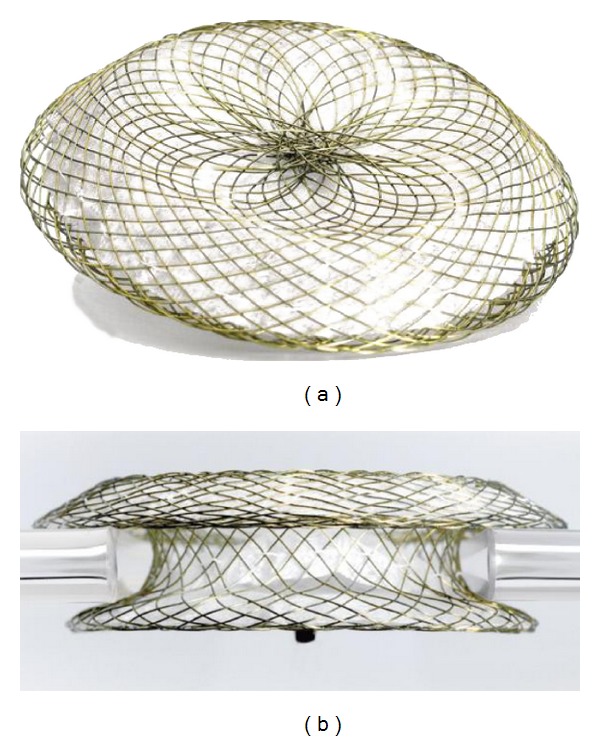
Occlutech Figulla^R^ ASD device.

The Occlutech Figulla^R^ device (Occlutech, Helsingborg, Sweden) (Figure 1) is a new device with increasing frequency and appears to be safe and effective to close secundum ASDs [10, 11]. This is the first report of transcatheter ASD closure in patient with ASD and wide opening fenestration of cor triatriatum by Occlutech Figulla^R^ device. 

## 2. Case Report

**Figure 2:**
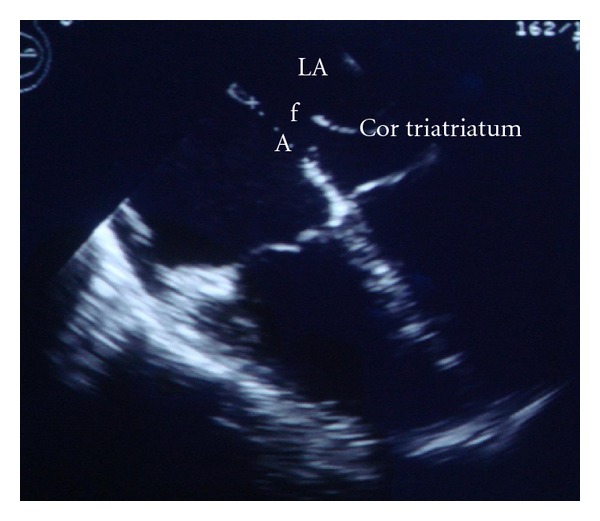
ASD secundum (A) and Cor triatriatum sinister with fenestration (f).

A 24-year-old male from the East of Thailand was found asymptomatic with functional class I and was found to have a heart murmur by routine checkup. He has no significant past medical history. Physical examination revealed a healthy man with height 176 cm and weight 60 kg. The Pulse rate was regular 60 bpm, blood pressure was 110/70 mmHg, and respiratory rate was 20 per min. Cardiac auscultation revealed a fixed split second sound with a grade 2/6 systolic ejection murmur at left upper parasternal border. Other systems were normal. Chest X-ray showed mild cardiomegaly with increase pulmonary blood flow. Transthoracic echocardiogram (TTE) and transesophageal echocardiogram(TEE) demonstrated right atrial and right ventricular enlargement with ASD secundum of 26 × 31 mm and cor triatriatrium with wide opening of 20 mm of fenestration as type A2 of Rodefeld classification [2]. 

(Figure 2) There was no evidence of turbulent flow through fenestration and pulmonary hypertension. There were adequate rims (length of rims more than 10 mm) around ASD secundum, then closure of ASD using the transcatheter approach was decided.

Transcatheter closure was performed with the patient under general anesthesia. Heparin 5,000 IU and cefazolin 1gram were administered intravenously before the procedure. Vascular access was obtained via the right femoral vein using 7-Fr sheath and the right femoral artery using 5-Fr sheath. TEE was performed. Right and left cardiac catheterization was performed and showed a pulmonary arterial pressure of 30/13 mmHg, Qp : Qs 2 : 1, and normal pulmonary venous drainage into the left atrium. 

**Figure 3:**
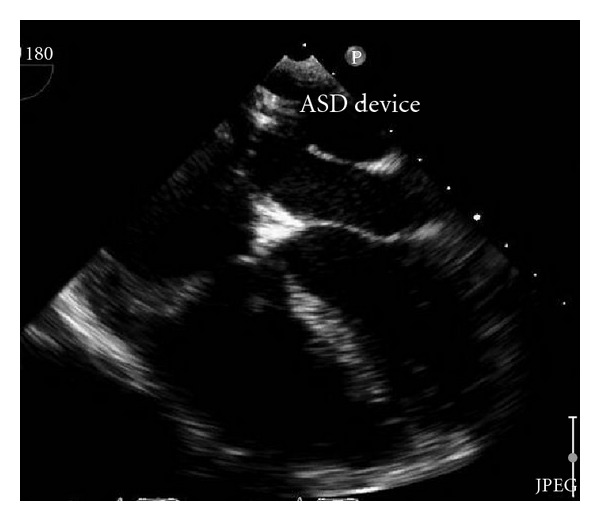
Occlutech Figulla device was deployed.

**Figure 4:**
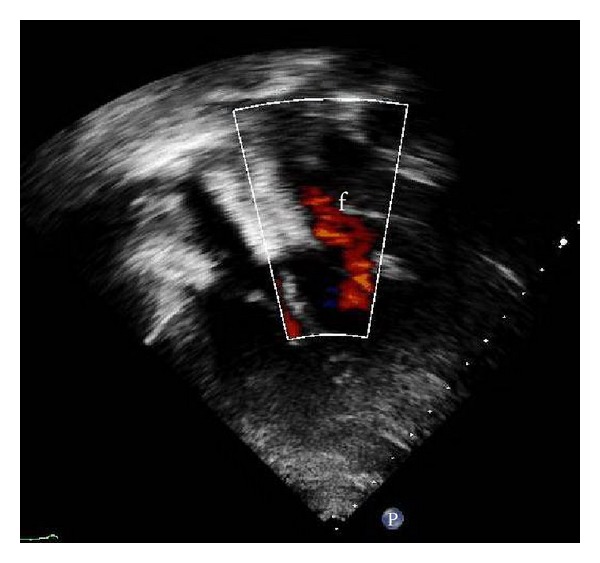
TEE after procedure demonstrate no obstruction blood flow through fenestration of cor triatriatum membrane (f).

**Figure 5:**
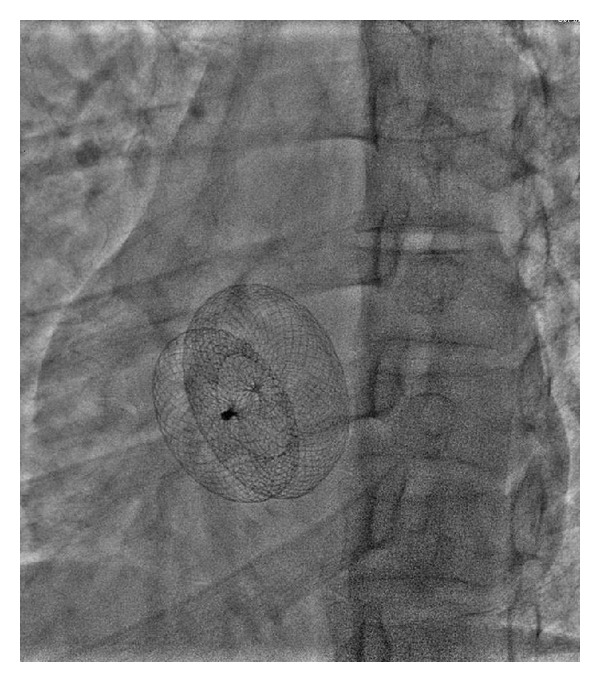
After releasing the device.

The first attempt for transcatheter closure using Cocoon^R^ ASD device (Vascular Innovations, Nonthaburi, Thailand) size 30 mm failed because we could not pull to deploy left atrial disc of device over atrial septum since cor triatriatum membrane was slipped into left atrial disc. Then we performed the second attempt using the Occlutech Figulla^R^ device. 6-Fr multipurpose (MP) catheter (Cook, Bloomington, USA) was placed in left upper pulmonary vein through ASD, then 0.035 Amplatz super stiff guide wire (Boston Scientific, Galway, Ireland) was inserted into MP catheter. PTS sizing balloon sizing (Numed, Cornwall, ON, Canada) 30 mm was inserted over super stiff guide wire. TEE demonstrated stretch diameter of ASD which was 31 mm by balloon sizing. 14-Fr Mullin's sheath (Cook, Bloomington, USA) was advanced through ASD over stiff guide wire. Occlutech Figulla^R^ device size 33 was loaded into delivery sheath and advanced by pusher cable to the left atrium. Device was deployed. (Figure 3) TEE revealed proper site of device without obstruction, no residual shunt, and residual fenestration of cor triatriatum membrane was 12 mm. We used TEE to recheck that the device will not occlude pulmonary venous return, and TEE revealed no turbulence flow of pulmonary veins and no turbulence flow of blood flow through fenestration of cor triatriatum membrane (Figure 4). Stability testing was performed. The device was released (Figure 5). No complication was found. TTE was performed in the next day and revealed the same result with TEE and no turbulence flow through fenestration of membrane. The patient was discharged with aspirin 300 mg once daily to be taken for 6 months.

Follow-up echocardiogram in the next 2, 6, and 12 months revealed the same result. The patient remained asymptomatic and no audible heart murmur was detected at auscultation. No complication was found.

## 3. Discussion

Cor triatriatum sinister with ASD in adults was corrected by surgical treatment in the previous studies [6, 12, 13]. All the surgical cases had inadequate opening of fenestration of cor triatriatum membrane. On the other hand, in this asymptomatic case with a wide opening of fenestration of cor triatriatum membrane, a suitable size of ASD, and adequate rim of tissue around ASD, transcatheter closure by ASD device closure is preferred over open surgery because it is less invasive and decrease hospital stay. The author's experience shows that size and position of device are the important things to recognize to avoid more obstruction of fenestration. Concerning the Occlutech Figulla^R^ device, the advantage of this device is the flatter profile of left atrial disc by absence of the left atrial disc microscrew (Figure 1), which decreases the obstruction to fenestration of cor triatriatum membrane in this case. The authors need to observe in the long term the side effects and complications.

In conclusion, transcatheter closure of ASD in patients with ASD and cor triatriatum sinister may be considered in patient with suitable size, adequate rims around ASD secundum, and a wide opening of fenestration of cor triatriatum membrane.
